# RETRACTED: Goud et al. Numerical Case Study of Chemical Reaction Impact on MHD Micropolar Fluid Flow Past over a Vertical Riga Plate. *Materials* 2022, *15*, 4060

**DOI:** 10.3390/ma17174388

**Published:** 2024-09-05

**Authors:** B. Shankar Goud, Yanala Dharmendar Reddy, Nawal A. Alshehri, Wasim Jamshed, Rabia Safdar, Mohamed R. Eid, Mohamed Lamjed Bouazizi

**Affiliations:** 1 Department of Mathematics, JNTUH College of Engineering Hyderabad Kukatpally, Hyderabad 500085, Telangana, India; bsgoud.mtech@gmail.com; 2 Department of Mathematics, Anurag University, Venkatapur, Hyderabad 500088, Telangana, India; dharmayanala@gmail.com; 3 Department of Mathematics, College of Science, Taif University, P.O. Box 11099, Taif 21944, Saudi Arabia; nalshehri@tu.edu.sa; 4 Department of Mathematics, Capital University of Science and Technology (CUST), Islamabad 44000, Pakistan; wasiktk@hotmail.com; 5 Department of Mathematics, Lahore College for Women University, Lahore 54000, Pakistan; rabia.safdar1109@gmail.com; 6 Department of Mathematics, Faculty of Science, New Valley University, El-Kharga 72511, Al-Wadi Al-Gadid, Egypt; 7 Department of Mathematics, Faculty of Science, Northern Border University, Arar 1321, Saudi Arabia; 8 Department of Mechanical Engineering, College of Engineering, Prince Sattam Bin Abdulaziz University, Alkharj 16273, Saudi Arabia; my.bouazizi@psau.edu.sa

The *Materials* Editorial Office retracts the article “Numerical Case Study of Chemical Reaction Impact on MHD Micropolar Fluid Flow Past over a Vertical Riga Plate” [1], cited above. 

Following publication, concerns were brought to the attention of the publisher regarding the validity of the parameters and equations presented in this article [1]. 

Adhering to our complaint procedure, an investigation was conducted by the Editorial Office and Editorial Board. While the authors fully cooperated with the Editorial Office during the investigation, they were unable to satisfactorily explain the presence of a number of significant anomalies within the parameters and the overall validity of the presented equations. Given the overall impact of these anomalies and validity concerns, the Editorial Board and Editor-in-Chief were unable to confirm the reliability of the findings and subsequently decided to retract this paper as per MDPI’s retraction policy (https://www.mdpi.com/ethics#_bookmark30, accessed on 29 May 2024) and in line with the Committee on Publication Ethics retraction guidelines (https://publicationethics.org/retraction-guidelines, accessed on 29 May 2024).

This retraction was approved by the Editor-in-Chief of the journal *Materials*. 

The authors disagree with this retraction. 

## References

[1] Goud B.S., Reddy Y.D., Alshehri N.A., Jamshed W., Safdar R., Eid M.R., Bouazizi M.L. (2022). RETRACTED: Numerical Case Study of Chemical Reaction Impact on MHD Micropolar Fluid Flow Past over a Vertical Riga Plate. Materials.

